# Hesperetin but not ellagic acid increases myosin heavy chain expression and cell fusion in C2C12 myoblasts in the presence of oxidative stress

**DOI:** 10.3389/fnut.2024.1377071

**Published:** 2024-09-02

**Authors:** Iris Cuijpers, Colin G. M. Dohmen, Freek G. Bouwman, Freddy J. Troost, Mireille M. J. P. E. Sthijns

**Affiliations:** ^1^Department of Human Biology, Institute of Nutrition and Translational Research in Metabolism (NUTRIM), Maastricht University, Maastricht, Netherlands; ^2^Food Innovation and Health, Centre for Healthy Eating and Food Innovation, Maastricht University Campus Venlo, Venlo, Netherlands

**Keywords:** polyphenols, hesperetin, ellagic acid, myoblasts, differentiation, oxidative stress

## Abstract

**Introduction:**

Skeletal muscle regeneration is impaired in elderly. An oxidative stress-induced decrease in differentiation capacity of muscle satellite cells is a key factor in this process. The aim of this study is to investigate whether orange polyphenol hesperetin and pomegranate polyphenol ellagic acid enhance myoblast differentiation in the presence and absence of oxidative stress, and to explore underlying mechanisms.

**Methods:**

C2C12 myoblasts were proliferated for 24 h and differentiated for 120 h while exposed to hesperetin (5, 20, 50 μM), ellagic acid (0.05, 0.1 μM) or a combination (20 μM hesperetin, 0.05 μM ellagic acid) with and without oxidative stress-inducing compound menadione (9 μM) during 24 h of proliferation and during the first 5 h of differentiation. The number of proliferating cells was assessed using fluorescent labeling of incorporated 5-ethynyl-2′-deoxyuridine. Myosin heavy chain expression was assessed by fluorescence microscopy and cell fusion index was calculated. Furthermore, protein expression of phosphorylated p38 and myomixer were assessed using Western blot.

**Results:**

None of the compounds induced effects on cell proliferation. Without menadione, 50 μM hesperetin increased fusion index by 12.6% compared to control (*p* < 0.01), while ellagic acid did not affect measured parameters of differentiation. Menadione treatment did not change myosin heavy chain expression and fusion index. In combination with menadione, 20 μM hesperetin increased myosin heavy chain expression by 35% (*p* < 0.01) and fusion index by 7% (*p* = 0.04) compared to menadione. Furthermore, the combination of menadione with hesperetin and ellagic acid increased myosin heavy chain expression by 35% compared to menadione (*p* = 0.02). Hesperetin and ellagic acid did not change p38 phosphorylation and myomixer expression compared to control, while treatment with menadione increased p38 phosphorylation (*p* < 0.01) after 5 h and decreased myomixer expression (*p* = 0.04) after 72 h of differentiation.

**Conclusion and discussion:**

Hesperetin increased myosin heavy chain expression in the presence of oxidative stress induced by menadione, and increased cell fusion both in the presence and absence of menadione. Ellagic acid did not affect the measured parameters of myoblast differentiation. Therefore, hesperetin should be considered as nutritional prevention or treatment strategy to maintain muscle function in age-related diseases such as sarcopenia. Future research should focus on underlying mechanisms and translation of these results to clinical practice.

## Graphical Abstract

Treatment with hesperetin during 120 h of differentiation increased myoblast fusion compared to control. Treatment with menadione or ellagic acid did not affect myosin heavy chain expression or cell fusion. In presence of oxidative stress, hesperetin treatment increased myosin heavy chain expression as well as fusion index compared to treatment with menadione alone. The combination of hesperetin and ellagic acid increased myosin heavy chain expression in cells exposed to oxidative stress. Treatment with oxidative stress inducing compound menadione for 5 h at the start of differentiation increased p38 expression at 5 h and decreased myomixer expression at 120 h of differentiation. Hesperetin (HSP), ellagic acid (EA), menadione (MEN), mitogen-activated protein kinase 14 (p38). Created with BioRender.com. 
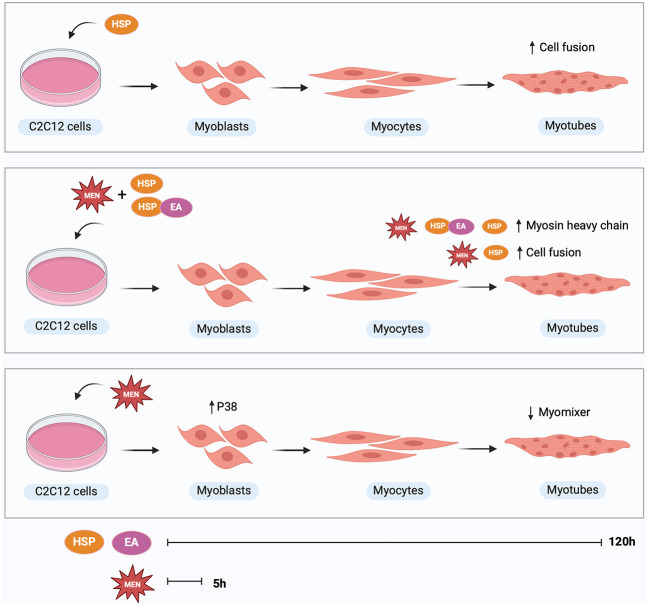

## 1 Introduction

With increasing age, skeletal muscle mass and function decrease progressively, resulting in reduced muscle strength, frailty, and poorer quality of life (1). This impairment in muscle function, also referred to as sarcopenia, is a common health problem affecting approximately 10–27% of the global population aged 60 years and older in 2019 (2). One of the factors contributing to development of sarcopenia is a decreased number and differentiation capacity of muscle stem cells (3, 4). Muscle stem cells, also referred to as satellite cells, are undifferentiated muscle progenitor cells that reside between the basal membrane and the plasma membrane of muscle fibers. When satellite cells get activated due to physical trauma or muscle strain, they proliferate, migrate as myoblasts to injured muscle tissue, differentiate, and fuse to repair the myofibers. A subset of satellite cells is able to self-renew and return to quiescence to replenish the stem cell pool and prepare for subsequent muscle damage (5). Myogenic differentiation is tightly regulated by various protein kinases and basic helix-loop-helix myogenic regulatory factors. Mitogen-activated protein kinase 14, also called p38α MAPK, which was initially identified as an effector of the cellular stress response, has shown to play a critical role during myoblast differentiation (6). Phosphorylation of p38α induces a powerful trigger for cell cycle exit by downregulation of proliferation markers (e.g., cyclins, retinoblastoma protein) (7). Additionally, phosphorylated p38α stimulates activation of myogenic transcription factors myoblast determination protein 1 (myoD) and myogenin, leading to maturation of muscle cells via increasing the expression of myosin heavy chain, a protein facilitating muscle contraction and relaxation (6, 8). MyoD and myogenin bind to the gene promoter of myomaker and myomixer proteins, which are membrane bound proteins that play an important role during muscle cell fusion (9). Myomaker and myomixer are synthesized in the nucleus and transported via vesicles to the muscle cell membrane, where myomaker is thought to be responsible for establishment of the hemifusion state and myomixer is involved in membrane pore formation during cell fusion (10).

Aged muscles show higher levels of reactive oxygen species (ROS), which is thought to be a major contributor to the deterioration of muscle regeneration with age (11, 12). Due to increased oxidative stress and decreased antioxidant enzymes, aged skeletal muscle is more vulnerable to oxidative stress induced damage (11, 13, 14). Physiological levels of ROS are needed for satellite cell activation which enhances phosphorylation of p38 and thereby induces the activation of the p38 MAPK pathway (15). However, excessive amount of ROS could lead to cellular damage and impaired functionality (16). Hydrogen peroxide (H_2_O_2_) exposure resulted in decreased myoblast proliferation and decreased expression of myosin heavy chain in myotubes, indicating an impaired differentiation capacity (17, 18). Besides H_2_O_2_, menadione is commonly used in *in vitro* cell models to induce oxidative stress by generating intracellular ROS at multiple cellular sites through futile redox cycling (19). As oxidative stress plays a major role in the age-related decline in muscle mass and function, a diet rich in polyphenols could be a promising intervention to combat this health problem (20). Consumption of a polyphenol rich diet has shown to be inversely related to the risk of many ROS-related diseases (21). Citrus polyphenol hesperidin (and its derivative hesperetin) and ellagitannins (hydrolyzed into ellagic acid) present in pomegranate and several berries, have shown great potential to oppose the age-related decrease in muscle health (22–25). Polyphenols including hesperetin and ellagic acid show strong antioxidant capacity in *in vitro* and rodent models by radical scavenging and upregulating levels of primary antioxidant enzymes, such as superoxide dismutase, catalase and glutathione peroxidase in lung and muscle tissue and in hepatic cells (22, 26–28). During scavenging of reactive oxygen species, antioxidants donate an electron or a hydrogen atom, thereby neutralizing the ROS and decreasing its activity (29). Hesperetin and ellagic acid can also augment cellular antioxidant defense capacity via nuclear translocation of Nrf2 and thereby increased expression of endogenous antioxidant enzymes (30, 31). Interestingly, hesperetin has shown to promote myogenic differentiation in mouse myoblasts and *ex vivo* muscle tissue through activation of MyoD and thereby enhancing myogenin gene expression (23). Furthermore, supplementation with pomegranate juice containing high concentrations of ellagitannins and ellagic acid, has shown to accelerate muscle recovery and decrease muscle soreness after strenuous bouts of eccentric exercise in trained men, suggesting a beneficial role in promoting muscle regeneration (24, 25).

As the role of ellagic acid during post-exercise muscle recovery is currently unknown, it is of interest to explore the effect of ellagic acid on myoblast differentiation. The combined antioxidant properties and myogenesis-enhancing potential of hesperetin and ellagic acid make this phenolic compound combination a promising intervention to address the age-related decline in skeletal muscle function. This study is the first to describe the effects of this phenolic compound combination on skeletal muscle differentiation. Additionally, this study expands current understanding on how muscle cells respond to hesperetin and ellagic acid, by investigating the effect of these polyphenols on the underlying mechanisms that are involved in myogenic differentiation (p38 phosphorylation) and cell fusion (myomixer). The aim of this study is to investigate whether treatment with hesperetin and ellagic acid affects myogenic differentiation of myoblasts with and without exposure to oxidative stress, and to explore the underlying mechanisms. It is hypothesized that hesperetin and ellagic acid can ameliorate the oxidative stress-induced decline in cell proliferation and differentiation, and enhance myogenic differentiation by increasing myosin heavy chain expression and cell fusion independently of oxidative stress.

## 2 Materials and methods

### 2.1 Chemicals

Hesperetin, ellagic acid, menadione sodium bisulfite, dimethyl sulfoxide (DMSO), sodium hydroxide (NaOH), 5-ethynyl-20-deoxyuridine (EdU)-Click 488 assay, formaldehyde, bovine serum albumin (BSA), Tween20 (immunofluorescence), 4′,6-Diamidino-2-phenylindole dihydrochloride (DAPI) staining solution, β-nicotinamide adenine dinucleotide reduced disodium salt hydrate (NADH), sodium pyruvate, Triton X-100, anti-MYH1 rabbit monoclonal antibody (Cat# ZRB1214, RRID:AB_3083662) and goat anti-rabbit Texas-Red secondary antibody (Cat# SAB3700888, RRID:AB_3083663) were purchased from Merk Life Sciences N.V. (Amsterdam, the Netherlands). Penicillin-streptomycin (*p*/s; 10,000 U/ml penicillin, 10,000 μg/ml streptomycin), N-2-hydroxyethylpiperazine-N-2-ethane sulfonic acid (HEPES), protease and phosphatase inhibitor (containing aprotinin, bestatin, E-64, leupeptin, sodium fluoride, sodium orthovanadate, sodium pyrophosphate, β-glycerophosphate), RIPA buffer [25 mM Tris-HCl pH 7.6, 150 mM NaCl, 1% NP-40 (v/v), 1% sodium deoxycholate (w/v), 0.1% SDS (w/v)], Laemmli sample buffer reducing 6x [375 mM Tris-HCl, 9% SDS (w/v), 50% Glycerol (v/v), 9% 2-mercaptoethanol (v/v), 0.03% Bromophenol blue (w/v)] and donkey anti-mouse Alexa Fluor 680 (Cat# A10038, RRID:AB_11180593) were obtained from ThermoFisher Scientific (Waltham, Massachusetts, USA). Phosphorylated (total extract from C-9 glioma cells treated with anisomycin at 25 μg/ml for 3 0 min) and non-phosphorylated (total extract from C-9 glioma cells) p38 MAP Kinase Control Cell Extracts, P38 MAPK rabbit monoclonal antibody (Cat# 8690, RRID:AB_10999090) and phospho-p38 MAPK mouse monoclonal antibody (Cat# 9216, RRID:AB_331296) were purchased from Cell Signaling Technology (Danvers, Massachusetts, USA). Intercept (PBS) blocking buffer and donkey anti-rabbit IRDye 800CW secondary antibody (Cat# 926-32213, RRID:AB_621848) were derived from LI-COR (Lincoln, Nebraska, USA). Embryonic stem cell and germ cell specific protein (ESPG) sheep polyclonal antibody (Cat# AF4580, RRID:AB_952042) was purchased from R&D Systems Inc. a Bio-Techne Brand (Minneapolis, Minnesota, USA). Donkey anti-sheep DyLight^TM^ 800 secondary antibody (Cat# 613-745-168, RRID:AB_1961680) was obtained from Rockland Immunochemicals (Philadelphia, Pennsylvania, USA). Tween20 (Western blotting) was purchased from Bio-Rad Laboratories (Hercules, California, USA).

### 2.2 Cell culture and treatments

C2C12 murine skeletal myoblasts (ATCC CRL-1772, Manassas, Virginia, USA) were cultured in growth medium containing low glucose (1 g/L) Dulbecco's modified Eagle medium (DMEM; ThermoFisher Scientific) supplemented with 9% fetal bovine serum (FBS; ThermoFisher Scientific) (v/v) and 1% *p*/s (v/v) (32). Cells were cultured in a humidified atmosphere containing 5% CO_2_ at 37°C and passaged when reaching 60–70% confluency. Passage numbers from 10 to 15 were used.

**Figure 1:**
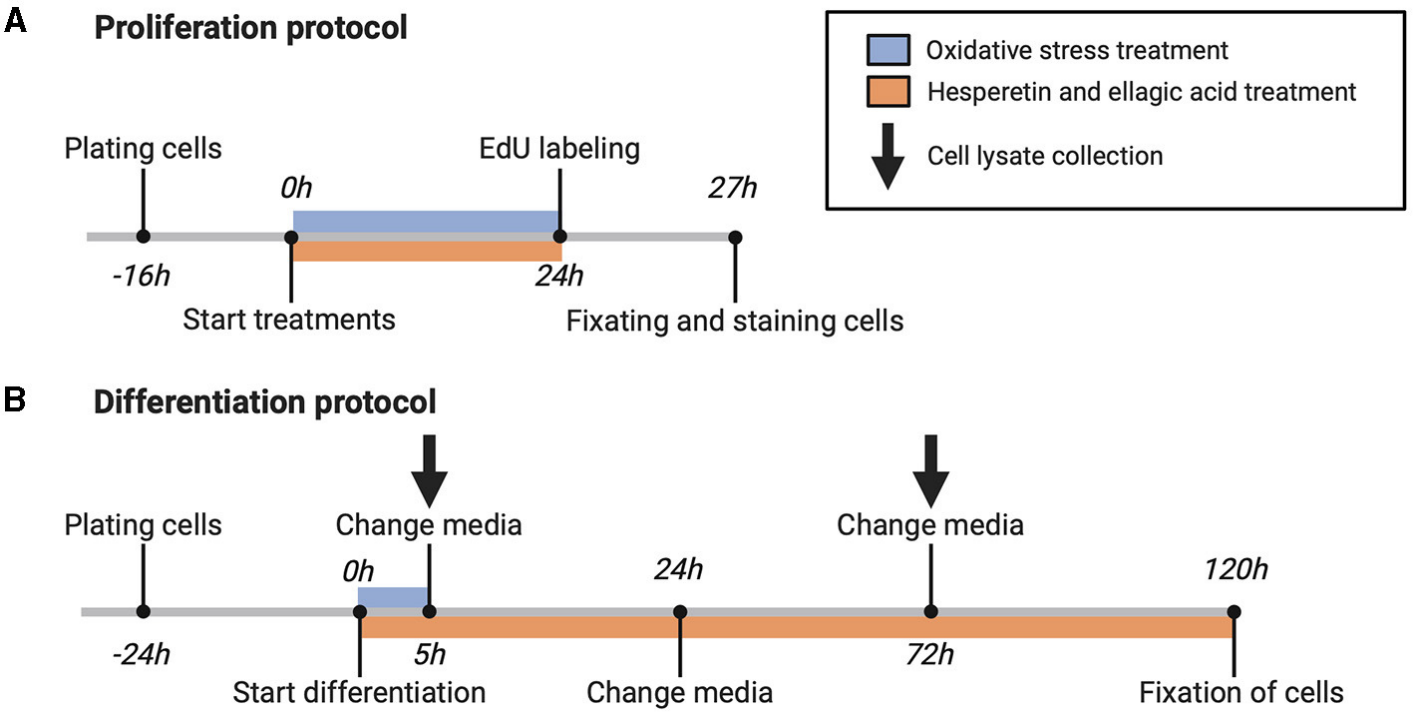
Schematic overview of proliferation **(A)** and differentiation **(B)** protocol. 5-ethynyl-20-deoxyuridine (EdU). Created with BioRender.com.

For cell proliferation measurement, cells (passage number 12–13) were seeded on Matrigel coated 24-well black culture plates (PerkinElmer, Waltham, Massachusetts, USA) at a density of 11,000 cells/well and were cultured for 16 h in growth medium. After reaching 40–50% confluency, cells were washed with Dulbecco's phosphate-buffered saline (DPBS; ThermoFisher Scientific) and incubated with growth medium containing hesperetin (5, 20, 50 μM), ellagic acid (0.05, 0.1 μM), or a combination of hesperetin (20 μM) and ellagic acid (0.05 μM) in the absence or presence of oxidative stress inducing compound menadione sodium bisulfite (9 μM) for 24 h (Figure 1A). DMSO and 0.5 M NaOH were used to dissolve hesperetin and ellagic acid, respectively (maximum concentration of 0.1% in final working solution (v/v)). Menadione sodium bisulfite was dissolved in distilled water. Growth medium without FBS was used as a negative control condition.

For assessment of C2C12 cell differentiation, cells (passage number 10–15) were seeded on Matrigel coated 24-well black or 6-well clear culture plates (Greiner Bio-One, Frickenhausen, Germany) at a density of 25,000 cells/well (for immunofluorescence experiments) and 125,000 cells/well (for Western blot experiments), respectively. Cells were grown for 24 h until 80–90% confluency was reached, followed by replacement of growth medium with differentiation medium containing high glucose (4.5 g/L) DMEM (ThermoFisher Scientific) supplemented with 1% heat inactivated FBS (hiFBS, ThermoFisher Scientific) (v/v) and 2.5% HEPES (v/v). Differentiation medium contained menadione sodium bisulfite, hesperetin and/or ellagic acid in the same concentrations and combinations as during cell proliferation experiments. Cells were treated with menadione during the first 5 h of differentiation, and after 5, 24, and 72 h differentiation medium was changed containing freshly prepared polyphenol solutions (Figure 1B).

### 2.3 Cell proliferation assay

C2C12 cell proliferation was assessed by an EdU-Click 488 assay. After 24 h of compound treatment, cells were incubated with 10 μM EdU for 3 h and fixed in 4% formaldehyde in DPBS (v/v) for 15 min. Cells were washed with a blocking buffer consisting of 3% BSA in DPBS (w/v) and permeabilized for 15 min using 0.2% Tween20 in DPBS (v/v) followed by 30 min incubation with reaction cocktail to detect the incorporated EdU. Afterwards, cells were stained with a DAPI nuclei staining (4 μM for 15 min). Images were obtained with a fluorescent microscope (EVOS FL, Thermo Fisher) and analysis was done using Fiji software version 1.53t. The StarDist plugin was used to calculate the percentage of EdU-positive cells from total number of nuclei stained with DAPI (33, 34).

### 2.4 Immunofluorescence staining

Differentiated cells were fixated in 4% formaldehyde in DPBS (v/v) for 15 min, washed with DPBS, and permeabilized in 0.2% Tween20 in DPBS (v/v) for 15 min. Thereafter, blocking was performed for 15 min using a solution of 1% BSA in DPBS (w/v) and cells were incubated with anti-MYH1 (myosin heavy chain type IIx, MYH1 gene) rabbit monoclonal primary antibody (0.3 μg/mL). After overnight incubation with primary antibody, cells were washed and incubated with goat anti-rabbit Texas-Red secondary antibody (4 μg/mL) for 45 min followed by a DAPI nuclei staining (4 μM for 15 min). An overview of antibodies used for immunofluorescence stainings is shown in Table 1. Images were obtained with a fluorescent microscope (EVOS FL, Thermo Fisher) and analyses were done using Fiji software. Mean intensity of myosin heavy chain fluorescent signal was corrected for the total number of nuclei and shown as fold change from the control condition. Fusion index was calculated as the number of nuclei inside myosin heavy chain positive myotubes (containing ≥2 nuclei) as a percentage of the total number of nuclei.

**Table 1:** Overview of primary and secondary antibodies used in the study.

**Primary antibodies**						
**Antigen**	**Host species**	**Species reactivity**	**Dilution factor**	**Supplier**	**#Cat**	**RRID**
MYH1 (MyHC IIx)	Rabbit	Human, mouse	1:100 (IF)	Merck Life Sciences	ZRB1214	AB_3083662
P38 MAPK	Rabbit	Human, mouse, rat, hamster, monkey, bovine, pig	1:1,000 (WB)	Cell Signaling Technology	8690	AB_10999090
Phospho-P38 MAPK	Mouse	Human, mouse, rat, monkey, *S.cerevisiae*	1:1,000 (WB)	Cell Signaling Technology	9216	AB_331296
ESPG (myomixer)	Sheep	Mouse	1:2,000 (WB)	R&D Systems	AF4580	AB_952042
**Secondary antibodies**						
**Conjugate**	**Host species**	**Species reactivity**	**Dilution factor**	**Supplier**	**#Cat**	**RRID**
Texas Red	Goat	Rabbit	1:500 (IF)	Merck Life Sciences	SAB3700888	AB_3083663
IRDye 800CW	Donkey	Rabbit	1:10,000 (WB)	LI-COR	926-32213	AB_621848
Alexa Fluor 680	Donkey	Mouse	1:10,000 (WB)	ThermoFisher Scientific	A10038	AB_11180593
DyLight 800	Donkey	Sheep	1:15,000 (WB)	Rockland Immunochemi-cals	613-745-168	AB_1961680

#Cat, Category number; RRID, Research Resource Identifier; (MYH, MyHC), myosin heavy chain; p38 MAPK, p38 mitogen-activated protein kinase; ESPG, embryonic stem cell and germ cell specific protein; IF, immunofluorescence; WB, Western blot.

### 2.5 Protein extraction and Western blotting

Cells were washed in ice-cold DPBS and lysed at indicated time points using RIPA lysis and extraction buffer (200 μL/well for a 6-well plate) in the presence of 1% protease and phosphatase inhibitor (v/v) (Figure 1B). Total protein content of the cell lysate was determined with a bicinchoninic acid assay (BCA, ThermoFisher Scientific). Five parts of sample were diluted with 1 part of Laemmli sample buffer, samples were boiled for 3 min at 100°C and shortly spinned using a microcentrifuge (14,000 rpm). Samples containing 10 μg of protein were loaded and seperated on polyacrylamide gel (8–16% Criterion TGX Stain-free Protein Gel, Bio-Rad Laboratories Inc.), and transferred using a trans-blot turbo nitrocellulose transfer pack (Bio-Rad Laboratories Inc.). Additionally, 15 μl of phosphorylated and non-phosphorylated p38 MAPK control cell extracts were loaded on each gel as a positive and negative control (35–37). After blocking non-specific binding sides in intercept blocking buffer for 1h, blots were incubated in anti-p38 MAPK rabbit monoclonal antibody (1:1,000), anti-Phospho-p38 MAPK mouse monoclonal antibody (1:1,000) and anti-ESPG sheep polyclonal antibody (0.1 μg/mL) overnight at 4°C. Blots were washed three times with PBS 0.1% Tween20 (PBST) (v/v) and incubated with donkey anti-mouse Alexa Fluor 680 (0.2 μg/mL), donkey anti-rabbit IRDye 800CW (0.1 μg/mL) and donkey anti-sheep DyLight^TM^ 800 (0.067 μg/mL) secondary antibody for 1 h at room temperature. An overview of antibodies used for Western blots is shown in Table 1. After secondary antibody incubation, blots were washed (twice in PBST and once in PBS) and near-infrared fluorescent images were made using the Odyssey CLx imaging system (LI-COR). Fluorescent bands were quantified using Image StudioTM software version 5.2 (LI-COR) and Stain-Free total protein normalization was performed using Image Lab software version 6.01 build 34 (Bio-Rad Laboratories Inc.). Data were shown as fold change from the control condition and phosphorylation of p38 was expressed by the level of phosphorylated (*p*)-p38 corrected for total amount of p38.

### 2.6 Statistical analyses

Data are presented as mean ± standard error of the mean (SEM), and if not normally distributed as median and interquartile range. Statistical analyses were performed with Graphpad Prism 10.1.0 software (GraphPad Software, Boston, MA, USA). Normality was checked with the Shapiro-Wilk test. Depending on the distribution of the data, a one-way ANOVA (for outcomes of cell proliferation, fusion index and myomixer in oxidative stress treated cells, (*p*)-p38/p38 5 h in cells without oxidative stress) or Kruskal-Wallis test (all other outcome measures) was used to test for statistically significance. At least three independent experiments were performed for each outcome measure. *N* corresponds to the number of independent experiments, while *n* indicates the number of replicates. *P*-values < 0.05 were considered statistically significant.

## 3 Results

### 3.1 Menadione, hesperetin and ellagic acid did not affect C2C12 proliferation

**Figure 2:**
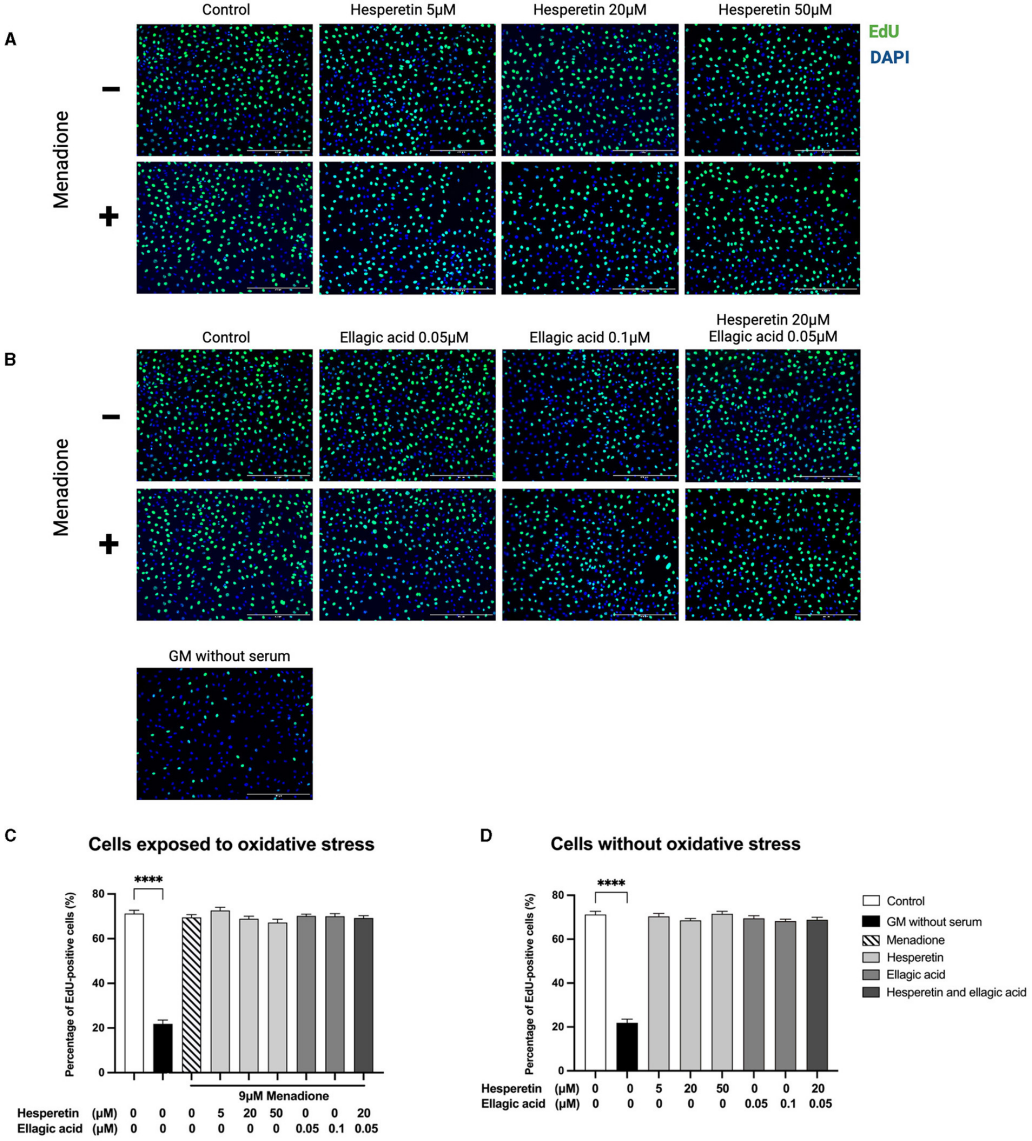
Immunofluorescence images of C2C12 exposed to hesperetin (5, 20, and 50 μM, **A**), ellagic acid (0.05 and 0.1 μM, **B**) or a combination of these (hesperetin 5 μM and ellagic acid 0.05 μM, **B**) for 24 h in the presence or absence of oxidative stress (9 μM menadione). Cells were stained with 5-ethynyl-20-deoxyuridine (EdU, green) and 4′,6-Diamidino-2-phenylindole dihydrochloride (DAPI) nuclei staining (blue). None of the compounds did affect C2C12 cell proliferation compared to control (*N* = 3, *n* = 2, including three images per well, **C, D**). Growth medium without serum was used as a negative control, which showed a significant reduction in cell proliferation compared to control **(C, D)**. Cell proliferation is shown as the percentage of EdU positive cells from the total number of cells. Data are presented as mean ± standard error of the mean (SEM). ****p < 0.0001. Created with BioRender.com.

Hesperetin (5, 20, or 50 μM), ellagic acid (0.05 or 0.1 μM) and the combination of hesperetin and ellagic acid (20 and 0.05 μM, respectively) in the presence or absence of oxidative stress inducing compound menadione (9 μM) did not affect cell proliferation (*p* > 0.99; Figure 2), while growth medium without FBS did decrease proliferation compared to control (*p* < 0.0001). As higher concentrations of hesperetin (100 μM) and ellagic acid (2.5, 50, 100 μM) induced cytotoxicity, they were not used for further experiments (Supplementary Figures 1, 2).

### 3.2 Hesperetin and the combination of hesperetin and ellagic acid increased myosin heavy chain expression in oxidative stress exposed C2C12 myotubes

**Figure 3:**
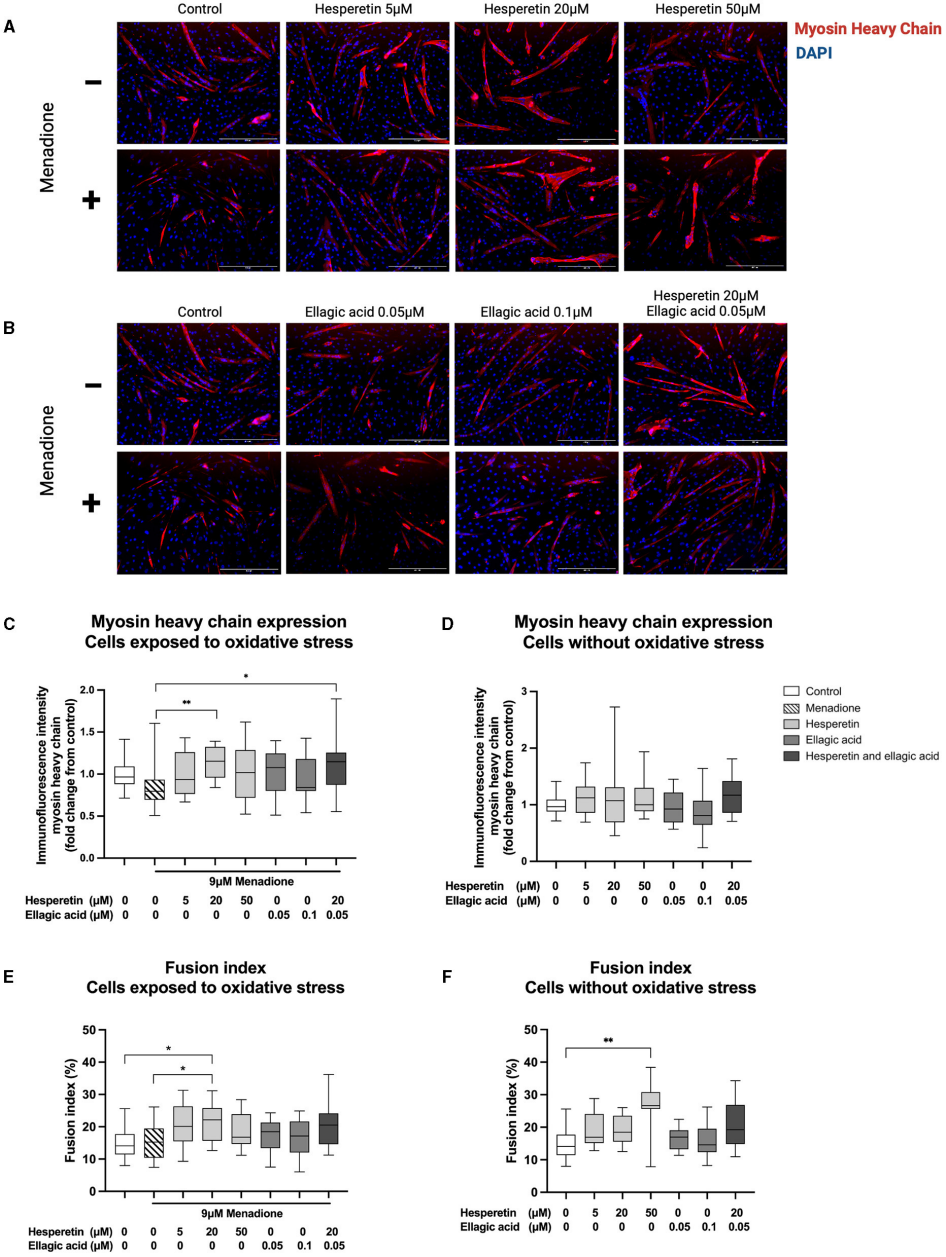
Immunofluorescence images of C2C12 cells after 5 days in differentiation media containing hesperetin (5, 20 and 50 μM; **A**), ellagic acid (0.05 and 0.1 μM; **B**) or a combination of these (hesperetin 5 μM and ellagic acid 0.05 μM; **B**) with and without exposure to oxidative stress inducing compound menadione (9 μM) in the first 5 h of differentiation. Cells were stained with myosin heavy chain type IIx (red) and 4′,6-diamidino-2-phenylindoledihydrochloride (DAPI) nuclei staining (blue). Treatment with 20 μM hesperetin and the combination of 20 μM hesperetin and 0.05 μM ellagic acid in oxidative stress exposed cells significantly increased the expression of myosin heavy compared to oxidative stress alone **(C)**. Hesperetin and ellagic acid did not affect myosin heavy chain expression compared to control **(D)**. Treatment with 20 μM hesperetin in C2C12 cells exposed to oxidative stress increased cell fusion compared to oxidative stress alone as well as control **(E)**. Additionally, treatment with 50 μM hesperetin increased fusion index compared to control **(F)**. Data were presented as median and quartiles. In figures **(C, D)**, immunofluorescence was corrected for the number of nuclei and shown relative to control. In figures **(E, F)**, fusion index was calculated as the number of nuclei inside myosin heavy chain positive myotubes (containing ≥ 2 nuclei) as a percentage of the total number of nuclei. Scale bar 400 μm. *p < 0.05, **p < 0.01, *N* = 3*, n* = 1 (including three images per well). Created with BioRender.com.

Immunofluorescence images revealed morphological changes after hesperetin treatment, with enlarged myosin heavy chain-positive structures compared to control and menadione-treated cells (Figures 3A, B). As a negative control, cells were stained with myosin heavy chain before the start of differentiation. This showed that myosin heavy chain was not expressed in myoblasts (data not shown). Treatment with 9 μM menadione showed a 20% decrease in myosin heavy chain expression compared to control, which was not statistically significant (0.80 (0.69–0.93), *p* = 0.39; Figure 3C). Furthermore, ellagic acid treatment in concentrations 0.05 and 0.1 μM did not affect myosin heavy chain expression compared to cells exposed to oxidative stress (*p* = 0.55 and *p* > 0.99, respectively; Figure 3C) as well as control cells (*p* > 0.99; Figure 3D). Treatment with 20 μM hesperetin in cells exposed to oxidative stress increased myosin heavy chain expression by 35% compared to oxidative stress alone (1.15 (0.96–1.32) vs. 0.80 (0.69–0.93), *p* < 0.01; Figure 3C). Similarly, the combination of 20 μM hesperetin and 0.05 μM ellagic acid increased myosin heavy chain expression by 35% compared to oxidative stress treated cells (1.15 (0.87–1.26) vs. 0.80 (0.69–0.93), *p* = 0.02; Figure 3C). Hesperetin treatment did not affect myosin heavy chain expression in control cells which were not exposed to oxidative stress (*p* < 0.99; Figure 3D). Descriptive data and *P*-values are shown in Supplementary Table 1.

### 3.3 Hesperetin increased cell fusion in C2C12 myotubes with and without exposure to oxidative stress

Treatment with 9 μM menadione did not lead to significant changes in fusion index compared to control (*p* = 0.99; Figure 3E). Furthermore, ellagic acid treatment in concentrations 0.05 and 0.1 μM and the combination of 20 μM hesperetin and 0.05 μM ellagic acid did not affect fusion index in C2C12 myotubes independently of exposure to oxidative stress (*p* = 0.96, *p* = 0.98 and *p* = 0.12, respectively, Figure 3E; *p* > 0.99, *p* > 0.99 and *p* = 0.17, respectively, Figure 3F). Treatment with 20 μM hesperetin in oxidative stress exposed cells significantly increased fusion index compared to oxidative stress alone (22.2% (15.6–25.8) vs. 15.2% (10.4–19.5), *p* = 0.04; Figure 3E) as well as control (22.2% (15.6–25.8) vs. 14.09% (11.4–17.7), *p* = 0.02; Figure 3E). Additionally, treatment with 50 μM hesperetin increased fusion index compared to control (26.7% (25.6–30.8) vs. 14.1% (11.4–17.7), *p* < 0.01; Figure 3F). Descriptive data and *P*-values are shown in Supplementary Table 2.

### 3.4 Treatment with menadione increased p38 phosporylation at 5 h and decreased myomixer expression at 72 h of differentiation

**Figure 4:**
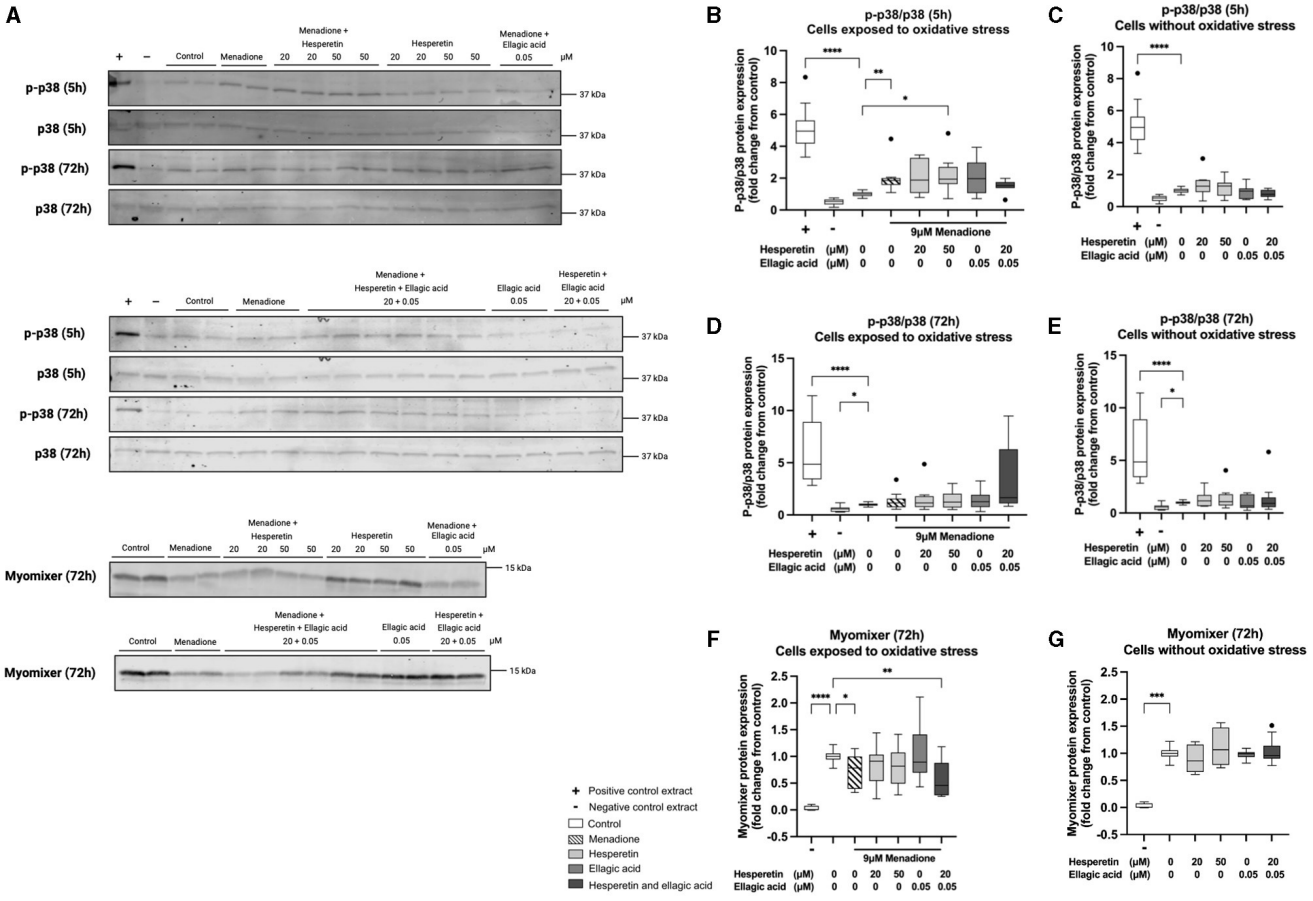
Western blots of p38 phosphorylation and myomixer expression in C2C12 cells that were lysed after 5 and 72 h in differentiation medium containing hesperetin (20 and 50 μM) ellagic acid (0.05 μM) or a combination of these (hesperetin 5 μM and ellagic acid 0.05 μM) with and without exposure to oxidative stress inducing compound menadione (9 μM) in the first 5 h of differentiation **(A)**. P38 phosphorylation was assessed by calculating the ratio of phosphorylated (*p*)-p38 and total p38 (*p*-p38/p38). Total extract from C-9 glioma cells with (+) and without (–) anisomycin treatment (25 μg/mL for 30 min) was used as positive and negative control. Five hour treatment with menadione in the absence and presence of 50 μM hesperetin increased p38 phosphorylation compared to control **(B)**, while the effect was gone after 72 h of differentiation **(D)**. Hesperetin (20 μM), Ellagic acid (0.05 and 0.1 μM) and the combination did not alter p38 phosphorylation after 5 and 72 h differentiation in C2C12 myotubes without exposure to oxidative stress **(C, E)**. As a negative control (–), myomixer expression was assessed before the start of differentiation, showing a significant decrease in myomixer expression compared to control **(F, G)**. Treatment with menadione in the absence and presence of the combination of hesperetin and ellagic acid significantly decreased the expression of myomixer compared to control **(F)**, while treatment with hesperetin and ellagic acid alone did not affect myomixer expression in C2C12 myotubes without oxidative stress exposure **(G)**. Data are presented as median and quartiles and shown relatively to control. *p < 0.05, **p < 0.01, ***p < 0.001, ****p < 0.0001. *N* = 4, *n* = 2. Created with BioRender.com.

Western blots are shown in Figure 4A. Five hour treatment with 9 μM menadione in absence (1.86 (1.58–2.00), *p* < 0.01) and presence of 50 μM hesperetin (1.94 (1.63–2.70), *p* = 0.02) increased p38 phosphorylation compared to control (Figure 4B), while the effect of menadione was gone after 72 h of differentiation (1.16 (0.74–1.57), *p* > 0.99, Figure 4D). Treatment with hesperetin (20, 50 μM), ellagic acid (0.05 μM) and the combination (hesperetin 20 μM and ellagic acid 0.05 μM) did not affect p38 phosphorylation at any of the time points in cells not exposed to oxidative stress (*p* > 0.05; Figures 4C, E). As a negative control, myomixer expression was assessed before the start of differentiation, showing a significant decrease in myomixer expression compared to control (0.0029 (−0.0017 to 0.076), *p* < 0.0001, Figure 4F). Menadione in absence (0.78 (0.39–1.00), *p* = 0.04) and presence of the combination of 20 μM hesperetin and 0.05 μM ellagic acid (0.46 (0.27–0.88), *p* = 0.002) decreased myomixer expression compared to control (Figure 4F), while treatment with hesperetin and ellagic acid did not alter myomixer expression in cells not exposed to oxidative stress (*p* > 0.05; Figure 4G). Descriptive data and *P*-values are shown in Supplementary Tables 3–5.

## 4 Discussion

This study investigated for the first time whether the combination of polyphenols hesperetin and ellagic acid enhance myogenic differentiation under oxidative stress conditions. Treatment of oxidative stress exposed cells with hesperetin, in a concentration of 20 μM, increased myosin heavy chain expression compared to myoblasts that were only exposed to oxidative stress. Treatment with a combination of 20 μM hesperetin and 0.05 μM ellagic acid in cells exposed to oxidative stress increased myosin heavy chain expression in a similar extend, suggesting that the combination did not result in a synergistic effect. As there was no effect of ellagic acid on myosin heavy chain expression in the presence of oxidative stress, this could also indicate that the effect shown by treatment with the combination of polyphenols is most likely due to the hesperetin treatment. Hesperetin treatment also increased fusion in cells that were not exposed to oxidative stress, while the combination of hesperetin and ellagic acid (with and without oxidative stress) did not alter the fusion index. This could suggest an antagonistic effect of ellagic acid, reducing the potential of hesperetin to enhance myocyte fusion. Treatment with menadione during the first 5 h did not lead to significant changes in C2C12 differentiation. Interestingly, treatment with menadione for 5 h increased p38 phosphorylation after 5 h of differentiation and decreased myomixer expression after 72 h of differentiation, while hesperetin and ellagic acid did not affect p38 phosphorylation or myomixer expression. Neither the oxidative stress-inducing compound menadione, nor any of the polyphenols hesperetin, ellagic acid separately or combined affected myoblast proliferation.

Treatment with hesperetin increased markers of myogenic differentiation. These effects were comparable to the study of Jeong et al., that found increased myosin heavy chain and myogenin protein expression when C2C12 cells were differentiated in the presence of 100 μM of hesperetin for 2–4 days (23). Myogenic differentiation could be enhanced due to the antioxidant effect or a direct effect of hesperetin on myogenic regulatory factors, as the same study showed that hesperetin promotes nuclear localization of myoD and its binding activity to the myogenin gene promoter (23). It was hypothesized that oxidative stress exposure inhibits myoblast differentiation. Excessive ROS levels in myoblasts could deplete intracellular antioxidants such as glutathione, which was shown in the study of Otten et al., in which 24 h treatment with 25 μM menadione decreased glutathione levels in C2C12 myotubes (38). ROS are also known to induce nuclear factor kappa B (NF-κB) signaling, consequently decreasing the expression levels of MyoD and myogenin, thereby inhibiting myogenic differentiation (39). Another study found decreased expression of myosin heavy chain in C2C12 that were exposed to 25 μM H_2_O_2_ for 72 h during differentiation (17). However, this study found no change in C2C12 differentiation after treatment with oxidative stress inducing compound menadione during the first 5 h of differentiation. The indirect stimulation of ROS production with menadione could have different or delayed effects in myoblasts as compared to direct ROS treatment using H_2_O_2_.

Treatment with ellagic acid in concentrations 0.05 and 0.1 μM did not affect myoblast differentiation in this study. While studies investigating the effects of ellagic acid on myogenic differentiation are lacking, one study found that 24 h-treatment with urolithin B (but not urolithin A), a product of ellagic acid catabolism, resulted in increased myotube diameter and fusion index in 4 days differentiated C2C12 cells (40). It has been demonstrated *in vivo* that end metabolites of ellagic acid, such as urolithin A and B, show a better ability to reach tissues and could therefore be responsible for the beneficial health effects of pomegranate supplementation (41). However, the production of urolithins is highly dependent on individual metabolic phenotypes, resulting in an extreme variability limiting the therapeutic potential of urolithins (41). In a bioavailability study, consumption of a single dose of pomegranate juice (180 mL containing 318 mg punicalagins and 12 mg of free ellagic acid) increased plasma levels of ellagic acid rapidly in all tested subjects (C_max_ = 0.06 μM, T_max_ = 0.98 h, T_1/2_ = 0.71 h), while detectable levels of urolithin A and B were only present in 2 out of 7 and 1 out of 7 subjects tested, respectively (42). Ellagic acid concentrations in plasma were comparable to the concentrations used in our cell experiments. Furthermore, alterations in fiber-type composition were seen in the back muscle of pigs and in muscles from mice following supplementation with ellagic acid, showing increased amount of slow-twitch muscle fibers and decreased levels of fast type myosin heavy chain (43, 44). Potentially, ellagic acid treatment increases levels of oxidative muscle fibers (slow type fibers) while in the current study, only type IIx myosin heavy chain fibers (fast type fibers) were assessed. Future research should focus on the effects of ellagic acid and urolithins on myogenic differentiation by assessment of multiple muscle fiber types. It should be considered whether a decrease in fast type fibers and an increase in slow type fibers induced by ellagic acid supplementation would be desirable in aging muscle, as the decrease in muscle mass and strength associated with sarcopenia is mainly caused by a decline in the number of fast type fibers (45).

To examine the underlying mechanism, the effects of menadione, ellagic acid and hesperetin on p38 phosphorylation and myomixer protein expression were determined. When phosphorylated, p38 stimulates MyoD activity by phosphorylating myogenic transcription factors myocyte-specific enhancer factor 2C (MEF2C) and transcription factor 3 (E47), that form a protein complex with MyoD before binding to target gene promotors such as myogenin, myosin heavy chain and membrane fusion protein myomixer (6, 8, 9, 46). Although menadione treatment did not result in significant changes in myosin heavy chain expression or fusion index, 5 h treatment with menadione at the start of differentiation increased the phosphorylation of p38 at 5 h and decreased myomixer expression at 72 h of differentiation. The increase in p38 phosphorylation following menadione treatment is in agreement with previous findings that show that the p38 MAPK pathway is activated in response to cellular stress (15). Increased p38 signaling in aged muscle has shown to result in slow and incomplete muscle repair. Aged satellite cells show intrinsically elevated p38 signaling, leading to an increased number of cells committed to differentiation at the expense of self-renewal (47). Hesperetin and ellagic acid did not affect p38 phosphorylation. While this is the first study investigating ellagic acid, the effect of hesperetin on p38 MAPK differs per cell type. In human glioblastoma cells, hesperetin induced apoptosis via activation of p38 MAPK (48). In a pancreatic cell line, hesperetin was used as a common p38 activator (49). Conversely, hesperetin has shown to inhibit p38 signaling in human umbilical vascular endothelial cells (50). Despite these effects found in other cell types, treatment with hesperetin did not alter p38 phosphorylation in C2C12 myotubes in this study, suggesting that other signaling pathways might be involved in the observed stimulating effect of hesperetin on myogenic differentiation. While it has been shown that increased oxidative stress inhibits myogenesis, only a limiting number of studies investigated the involvement of membrane protein myomixer (51, 52). Zocchi et al. showed that treatment with low and high concentrations of magnesium downregulated the expression of myomixer compared to physiological magnesium concentrations (52). This was prevented when cells were treated with N-Acetylcysteine and ROS production was inhibited, indicating that ROS were responsible for the impairment in myomixer expression. Gene knock-out experiments uncovered the crucial function of myomixer during myoblast differentiation and fusion. Deletion of myomixer in human myoblasts caused major defects in fusion, as the cells remained mononucleated after full-term differentiation (9). Hesperetin and ellagic acid did not affect myomixer expression, nor prevented the decline in myomixer expression induced by menadione.

Myoblast proliferation is an important step preceding myogenic differentiation and serves to increase the number of muscle progenitor cells. While low concentrations of oxidants have shown to stimulate mammalian cell proliferation, increased levels of ROS result in lower proliferation potential (53). Muscle satellite cells derived from glutathione peroxidase 1 deficient mice show decreased proliferation capacity compared to wild-type cells (54). Furthermore, exposure to H_2_O_2_ (at 1 mM for 30 min) resulted in an inhibition of proliferation of isolated human myoblasts (18). Our results are not in agreement with these studies, as treatment with oxidative stress inducing compound menadione for 24 h did not affect C2C12 cell proliferation. However, 24 h treatment with menadione in a concentration of 9 μM should be sufficient to increase mitochondrial ROS production in C2C12 cells (55). However, ROS produced in the mitochondria needs to reach the cell nucleus to be able to influence targets of cell proliferation. Additionally, treatment with hesperetin and ellagic acid did not affect C2C12 proliferation, while in other cell types inhibitory effects of hesperetin on cell proliferation (breast and skin carcinoma cells and rat aortic vascular smooth muscle cells) have been seen (56–58). Also ellagic acid has anti-proliferating properties in other cell lines (e.g., cancer cells from colon, breast and prostate), but no effect on myoblast proliferation has been observed in this study, using concentrations of 0.05 and 0.1 μM (59). A potential explanation why both hesperetin and ellagic acid did not affect cell proliferation is the higher level of endogenous antioxidant glutathione in skeletal muscle cells compared to breast cancer cells (60, 61).

It should be noted that the cell line used in this study, a C2C12 murine myoblast cell line, relies heavily on anaerobic glycolysis. Skeletal muscle cell lines which depend more on aerobic substrate utilization, such as L6 cells, may respond differently to oxidative stress and could show different effects in redox sensitive signaling pathways involved in myogenic differentiation (62). However, commonly used C2C12 cells (but also primary human myoblasts) better represent differentiated muscle cells in terms of myosin content and glycogen storage when compared to L6 myotubes, making these cell lines the preferred model for studies investigating effects on myoblast differentiation and its underlying mechanism (62). Primary human myoblasts show variation in cell cycle progression and overal transcriptome profile due to interindividual differences between donors (62). Investigations to the underlying mechanisms related to cell fusion, which involves assessing the expression of genes and proteins, are recommended to be conducted using C2C12 cells (63). When assessing the effects of hesperetin on regeneration, it is essential to also assess the total pool of muscle stem cells. Myoblast differentiation could contribute to repair of the oxidative stress-induced muscle damage. However, the absence of self-renewing of differentiated satellite cells could be detrimental for the overall regenerative capacity of the skeletal muscle. *In vivo* studies are necessary to investigate the maintenance of the satellite cell pool and cell senescence following hesperetin supplementation. Currently, available animal models for sarcopenia and exercise are rodent models, *Drosophila*, and *C. elegans*, but these animal models differ in their metabolism and uptake and therefore bioavailability of hesperetin and ellagic acid (64–66). Additionally, *Drosophila* and *C. elegans* have a different anatomy, differ physiologically in fast- and slow-contracting muscle (asynchronous and synchronous in *Drosophila*) and do not have any muscle satellite cells (66, 67). Skeletal muscle biopsies of aging individuals with and without supplementation with hesperetin could help to validate mechanistic effects of hesperetin by assessing protein and gene expression of myogenic regulatory factors and other key signaling factors involved in myogenic differentiation. Markers such as fibroblast growth factor (FGF), p38, cyclin-dependent kinase inhibitor 2A (p16^INK4A^) or senescence-associated beta-galactosidase (SA-β-gal) could be assessed to investigate the maintenance of the satellite cell pool and cell senescence. Additionally, it should be determined whether enhanced differentiation is associated with an increase in muscle function during long-term supplementation with hesperetin. Concentrations found in human plasma after supplementation with hesperetin (135 mg hesperetin, C_max_ = 2.73 μM, T_max_ = 3.7 h, T_1/2_ = 3.1 h) or after orange juice consumption (8 mL/kg, total of 126 ± 26 mg hesperetin, C_max_ = 2.20 μM, T_max_ = 5.4 h, T_1/2_ = 2.2 h) are comparable to concentrations used for cell experiments (68, 69). Poor bioavailablity of hesperetin can limit its effectiveness *in vivo* and methods enhancing the bioavailability of hesperetin should be evaluated. For example, a study of Takumi et al. shows an increase in maximum plasma concentrations of hesperetin with 5.5 times (C_max_ = 10.2 μM) by developing water-dispersible hesperetin by the process of micronization (70).

To conclude, this study showed that hesperetin increases myosin heavy chain expression in the presence of oxidative stress-inducing menadione and increases cell fusion both in the presence and absence of menadione. Ellagic acid did not affect the measured parameters of myoblast differentiation. Therefore, after validation of the effect of hesperetin on muscle function, it should be considered in nutritional prevention and treatment strategies of age-related decreases in muscle function such as in sarcopenia.

## Data availability statement

The original contributions presented in the study are publicly available. The data can be found here on DataverseNL: https://doi.org/10.34894/5Y0JTQ.

## Ethics statement

Ethical approval was not required for the studies on animals in accordance with the local legislation and institutional requirements because only commercially available established cell lines were used.

## Author contributions

IC: Conceptualization, Formal analysis, Investigation, Methodology, Visualization, Writing – original draft, Writing – review & editing. CD: Software, Writing – review & editing. FB: Methodology, Resources, Writing – review & editing. FT: Conceptualization, Funding acquisition, Resources, Supervision, Writing – review & editing. MS: Conceptualization, Methodology, Supervision, Writing – review & editing.
