# Supplementary material for: Hesperetin but not ellagic acid increases myosin heavy chain expression and cell fusion in C2C12 myoblasts in the presence of oxidative stress
Source: Front Nutr. 2024 Sep 2;11:1377071. doi: 10.3389/fnut.2024.1377071 (PMC11402829; doi:10.3389/fnut.2024.1377071)
Supplement: Supplementary file 1 [file Data_Sheet_1.docx]

Supplementary Material

# Supplementary Tables

## Supplementary Table 1

| **Myosin heavy chain expression (immunofluorescence intensity)** | | | |
| --- | --- | --- | --- |
| **Condition** | **Median and quartiles**  **(Fold change from control)** | **P-value**  **(compared to control)** | **P-value (compared to menadione)** |
| Control | 0.97 (0.88-1.09) | - | 0.39 |
| Menadione 9μM* | 0.80 (0.69-0.93) | 0.39 | - |
| Menadione 9μM + hesperetin 5μM | 0.94 (0.76-1.26) | >0.99 | 0.77 |
| Menadione 9μM + hesperetin 20μM | 1.15 (0.96-1.32) | >0.99 | **<0.01** |
| Menadione 9μM + hesperetin 50μM | 1.02 (0.72-1.29) | >0.99 | 0.68 |
| Menadione 9μM + ellagic acid 0.05μM | 1.08 (0.80-1.25) | >0.99 | 0.55 |
| Menadione 9μM + ellagic acid 0.01μM | 0.84 (0.80-1.18) | >0.99 | >0.99 |
| Menadione 9μM + hesperetin 20μM + ellagic acid 0.05μM | 1.15 (0.87-1.26) | >0.99 | **0.02** |
| Hesperetin 5μM | 1.13 (0.86-1.32) | >0.99 | - |
| Hesperetin 20μM* | 1.07 (0.69-1.31) | >0.99 | - |
| Hesperetin 50μM* | 1.00 (0.89-1.30) | >0.99 | - |
| Ellagic acid 0.05μM | 0.93 (0.69-1.22) | >0.99 | - |
| Ellagic acid 0.01μM | 0.81 (0.65-1.07) | >0.99 | - |
| Hesperetin 20μM + ellagic acid 0.05μM | 1.17 (0.86-1.42) | >0.99 | - |
| 0.1% DMSO | 0.84 (0.65-1.01) | 0.82 | - |
| 0.1% NaOH | 0.91 (0.58-1.20) | >0.99 | - |
|  |  |  |  |

**Supplementary Table 1.** Overview of median (quartiles) and P-values from myosin heavy chain expression data. Data were analysed using a Kruskal-Wallis test. * Data were not normally distributed. DMSO (dimethyl sulfoxide), NaOH (sodium hydroxide).

## Supplementary Table 2

| **Fusion index** | | | |
| --- | --- | --- | --- |
| **Condition** | **Median and quartiles**  **(%)** | **P-value (compared to control)** | **P-value (compared to menadione)** |
| Control | 14.1 (11.4-17.7) | - | 0.99 |
| Menadione 9μM | 15.2 (10.4-19.5) | 0.99 | - |
| Menadione 9μM + hesperetin 5μM | 20.1 (15.5-26.3) | 0.09 | 0.16 |
| Menadione 9μM + hesperetin 20μM | 22.2 (15.6-25.8) | **0.02** | **0.04** |
| Menadione 9μM + hesperetin 50μM | 16.8 (14.7-23.9) | 0.49 | 0.68 |
| Menadione 9μM + ellagic acid 0.05μM | 18.5 (13.4-21.3) | 0.85 | 0.96 |
| Menadione 9μM + ellagic acid 0.01μM | 17.2 (12.0-21.6) | 0.88 | 0.98 |
| Menadione 9μM + hesperetin 20μM + ellagic acid 0.05μM | 20.5 (14.6-24.2) | 0.06 | 0.12 |
| Hesperetin 5μM | 16.9 (15.2-24.1) | 0.39 | - |
| Hesperetin 20μM | 18.5 (15.6-23.5) | 0.21 | - |
| Hesperetin 50μM* | 26.7 (25.6-30.8) | **<0.01** | - |
| Ellagic acid 0.05μM | 17.0 (13.3-19.1) | >0.99 | - |
| Ellagic acid 0.01μM | 14.6 (12.4-19.5) | >0.99 | - |
| Hesperetin 20μM + ellagic acid 0.05μM | 19.2 (14.8-26.8) | 0.17 | - |
| 0.1% DMSO | 10.1 (6.3-13.8) | 0.40 | - |
| 0.1% NaOH | 15.1 (12.0-17.7) | >0.99 | - |

**Supplementary Table 2.** Overview of median (quartiles) and P-values from fusion index data. Fusion index was calculated as the number of nuclei inside MyHC-positive myotubes (containing ≥2 nuclei) as a percentage of the total number of nuclei. Data of menadione exposed conditions were analysed using a one-way ANOVA and conditions without oxidative stress were analysed using a Kruskal-Wallis test. * Data were not normally distributed. DMSO (dimethyl sulfoxide), NaOH (sodium hydroxide).

## Supplementary Table 3

| **p-p38/p38 5h (protein expression)** | | | |
| --- | --- | --- | --- |
| **Condition** | **Median and quartiles**  **(fold change from control)** | **P-value (compared to control** | **P-value (compared to menadione)** |
| Positive control | 4.95 (4.17-5.62) | **<0.0001** | 0.30 |
| Negative control | 0.54 (0.36-0.66) | 0.26 | **<0.0001** |
| Control | 1.00 (0.88-1.11) | - | **0.01** |
| Menadione 9μM* | 1.86 (1.58-2.00) | **<0.01** | - |
| Menadione 9μM + hesperetin 20μM | 1.88 (1.07-3.27) | 0.08 | >0.99 |
| Menadione 9μM + hesperetin 50μM | 1.94 (1.63-2.70) | **0.02** | >0.99 |
| Menadione 9μM + ellagic acid 0.05μM | 1.97 (1.06-2.98) | 0.10 | >0.99 |
| Menadione 9μM + hesperetin 20μM + ellagic acid 0.05μM | 1.53 (1.38-1.76) | 0.50 | >0.99 |
| Hesperetin 20μM | 1.28 (0.91-1.67) | 0.48 | - |
| Hesperetin 50μM | 1.29 (0.69-1.49) | 0.94 | - |
| Ellagic acid 0.05μM | 0.97 (0.48-1.13) | 0.99 | - |
| Hesperetin 20μM + ellagic acid 0.05μM | 0.77 (0.61-1.05) | 0.95 | - |

**Supplementary Table 3.** Overview of median (quartiles) and P-values from p-p38/p38 (5h) data. Data of menadione exposed conditions were analysed using a Kruskal-Wallis test and conditions without oxidative stress were analysed using a one-way ANOVA. * Data were not normally distributed.

## Supplementary Table 4

| **p-p38/p38 72h (protein expression)** | | | |
| --- | --- | --- | --- |
| **Condition** | **Median and quartiles**  **(fold change from control)** | **P-value (compared to control** | **P-value (compared to menadione)** |
| Positive control | 4.86 (3.42-8.91) | **<0.0001** | **0.01** |
| Negative control | 0.57 (0.30-0.71) | **0.05** | **<0.05** |
| Control | 1.00 (0.90-1.09) | - | >0.99 |
| Menadione 9μM* | 1.16 (0.74-1.57) | >0.99 | - |
| Menadione 9μM + hesperetin 20μM* | 1.16 (0.76-1.79) | >0.99 | >0.99 |
| Menadione 9μM + hesperetin 50μM | 1.22 (0.71-2.02) | >0.99 | >0.99 |
| Menadione 9μM + ellagic acid 0.05μM | 1.27 (0.76-1.94) | >0.99 | >0.99 |
| Menadione 9μM + hesperetin 20μM + ellagic acid 0.05μM* | 1.66 (1.11-6.29) | 0.07 | 0.74 |
| Hesperetin 20μM | 1.16 (0.65-1.71) | >0.99 | - |
| Hesperetin 50μM* | 1.09 (0.72-1.79) | >0.99 | - |
| Ellagic acid 0.05μM | 0.68 (0.49-1.79) | >0.99 | - |
| Hesperetin 20μM + ellagic acid 0.05μM* | 0.92 (0.56-1.49) | >0.99 | - |

**Supplementary Table 4.** Overview of median (quartiles) and P-values from p-p38/p38 (72h) data. Data were analysed using a Kruskal-Wallis test. * Data were not normally distributed.

## Supplementary Table 5

| **Myomixer 72h (protein expression)** | | | |
| --- | --- | --- | --- |
| **Condition** | **Median and quartiles**  **(fold change from control)** | **P-value (compared to control)** | **P-value (compared to menadione)** |
| Negative control | 0.0029 (-0.0017-0.076) | **<0.0001** | **<0.0001** |
| Control | 1.00 (0.94-1.06) | - | **0.04** |
| Menadione 9μM | 0.78 (0.39-1.00) | **0.04** | - |
| Menadione 9μM + hesperetin 20μM | 0.91 (0.54-1.03) | 0.48 | 0.90 |
| Menadione 9μM + hesperetin 50μM | 0.82 (0.49-1.07) | 0.39 | 0.94 |
| Menadione 9μM + ellagic acid 0.05μM | 0.89 (0.70-1.41) | >0.99 | 0.08 |
| Menadione 9μM + hesperetin 20μM + ellagic acid 0.05μM | 0.46 (0.27-0.88) | **0.002** | 0.71 |
| Hesperetin 20μM | 0.86 (0.66-1.16) | 0.79 | - |
| Hesperetin 50μM | 1.07 (0.79-1.48) | >0.99 | - |
| Ellagic acid 0.05μM | 0.99 (0.93-1.02) | >0.99 | - |
| Hesperetin 20μM + ellagic acid 0.05μM* | 0.96 (0.90-1.14) | >0.99 | - |

**Supplementary Table 5.** Overview of median (quartiles) and P-values from myomixer data. Data of menadione exposed conditions were analysed using a one-way ANOVA and conditions without oxidative stress were analysed using a Kruskal-Wallis test. * Data were not normally distributed.

# Supplementary Figure 1


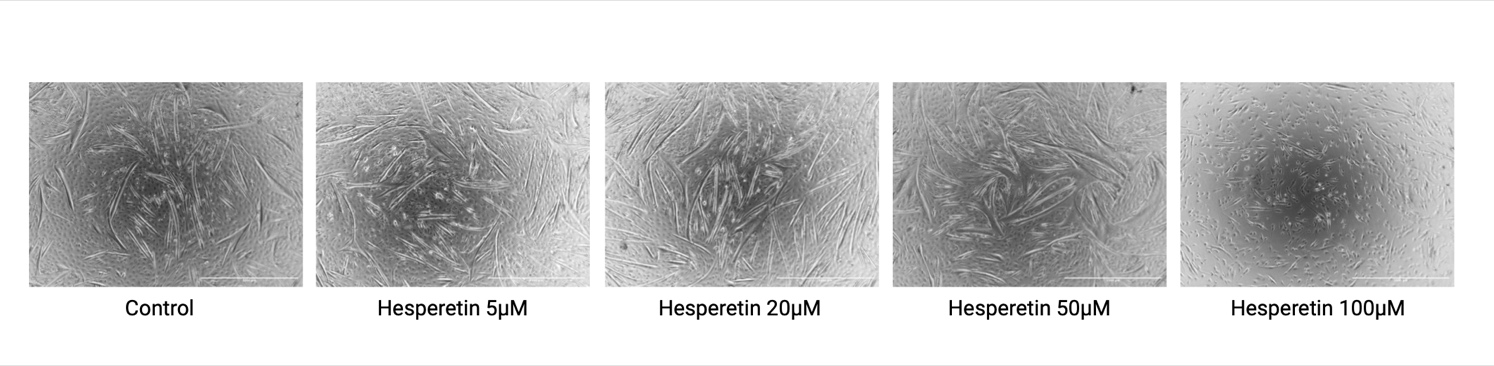


**Supplementary Figure 1.** Brightfield images of C2C12 cells that were exposed to different concentrations of hesperetin (5, 20, 50, 100μM) during 5 days of differentiation. Scalebar = 1000μm.


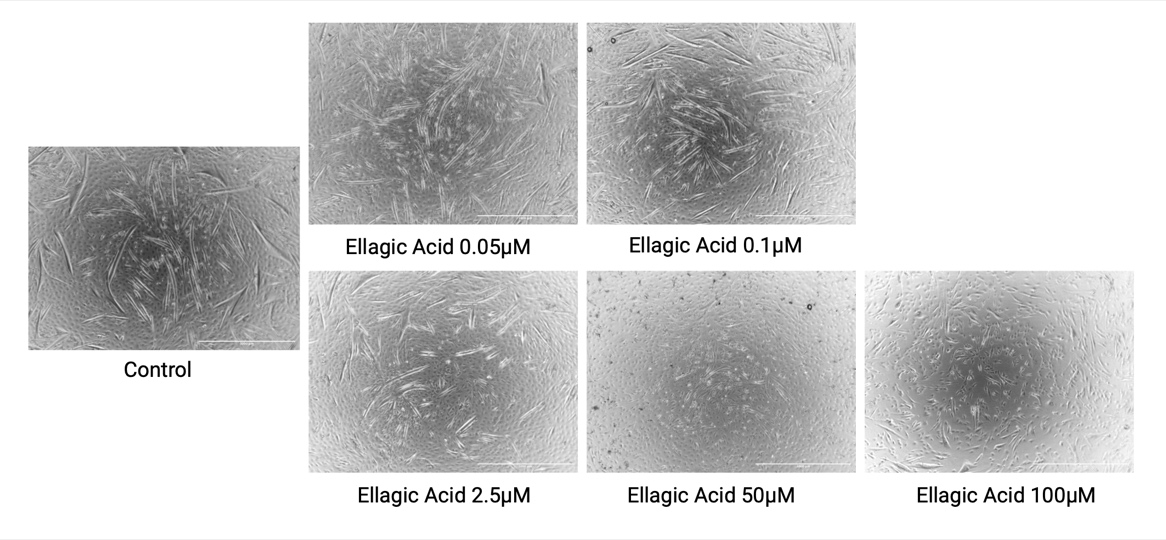


**Supplementary Figure 2.** Brightfield images of C2C12 cells that were exposed to different concentrations of ellagic acid (0.05, 0.1, 2.5, 50, 100μM) during 5 days of differentiation. Scalebar = 1000μm.
